# Supplementary material for: Injectable Carrageenan/Green Graphene Oxide Hydrogel: A Comprehensive Analysis of Mechanical, Rheological, and Biocompatibility Properties
Source: Polymers (Basel). 2024 Aug 19;16(16):2345. doi: 10.3390/polym16162345 (PMC11359936; doi:10.3390/polym16162345)
Supplement: Supplementary file 1 [file polymers-16-02345-s001.zip › polymers-3097145-supplementary.pdf]

Supplementary file

Innovative injectable hydrogel system combining carrageenan and green graphene oxide for advanced regenerative medicine.

Danny Moncada, Maite Rico, Saddys Rodríguez-Llamazares, Natalia Pettinelli, Alana Aragón-Herrera, Sandra Feijoo-Bandín, Oreste Gualillo, Francisca Lago, Yousof Farrag, Rebeca Bouza, Horacio J. Salavagione*

Supplementary Materials: The following supporting information can be downloaded at: www.mdpi.com/xxx/s1, Figure S1: TGA of GO comparing its behavior under an oxygen atmosphere with a nitrogen atmosphere; Figure S2: Raman spectra of GO and the GO (0.5% *w/v*) dispersed in ιC (2% *w/v*); Figure S3: spectra of the graphene (0.5%) suspension in ιC (2%) applying different ultrasonication times (5 min and 10 min); Figure S4: analysis of XRD patterns of injectable hydrogels (IH/IH+GO).

Supplementary Figures:

Figure S1: Characterization of GO

The thermal stability of the GO under nitrogen and oxygen atmospheres is shown in **Figure S1**. In the inert atmosphere, the GO decomposition occurs in one-step in a temperature range of 50 ºC – 800 ºC, with a loss of about 10 % by weight of GO. This weight loss is associated to decarboxylation, decarbonylation and dehydration of GO and is close to the expected value,[47] according to the oxygen content reported by the supplier. However, when GO is exposed to an oxygen atmosphere, the thermogram shows two processes occurring at temperatures very close to each other, one at 332 ºC and the other at 382 ºC, corresponding to the loss of oxygen functional groups present in the GO sheets and subsequent combustion of the carbon structure of graphene, respectively.[18,48,49] The residual analysis of the graphene content was close to 5%. The weight loss at temperatures below 300 ºC is very low (5%), so it can be said that it is very stable at the working temperature.

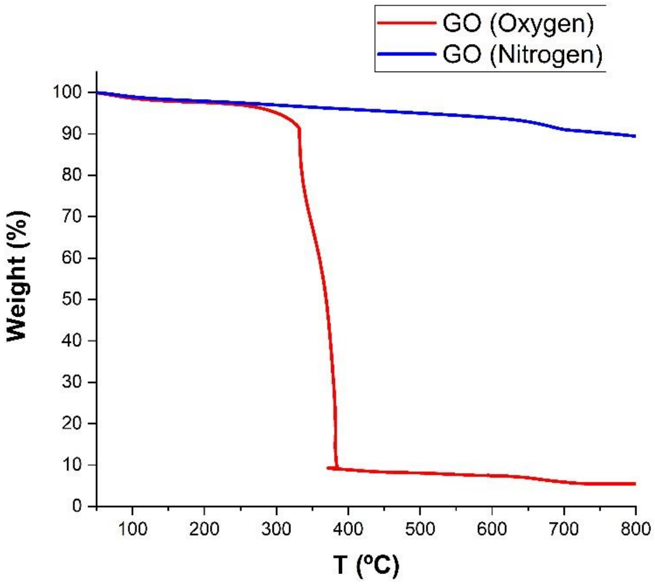

Figure S1. TGA of GO comparing its behavior under an oxygen atmosphere with a nitrogen atmosphere.

Figure S2: Raman spectra of the GO and ιC/GO samples

The Raman spectra of the GO and ιC/GO samples are shown in **Figure S2**. The Raman spectrum shows two characteristic peaks of graphene oxide, the G band around 1595 cm^{-1}, associated to the vibration of carbon atoms with sp^2 bonds in a two-dimensional hexagonal lattice and the known-as D band. around 1319 cm^{-1},[18] corresponding to the vibrations of sp^3 hybridized carbon atoms representing imperfections in the graphene structure, and. According to previous studies, disorder in graphene nanosheets can be originated from defects associated with vacancies, grain boundaries and amorphous carbon species.[26]

The I_D/I_G ratio, which reflects the density of defect over degree of graphitization of carbonaceous materials similar gives of 2.4 and 2.7 for solid GO and ιC/GO, respectively.[26] The high I_D/IG values are somewhat expected due to the intrinsically defective GO structure and can also have a contribution from the ultrasound process used to enhance the dispersion.[48–50]

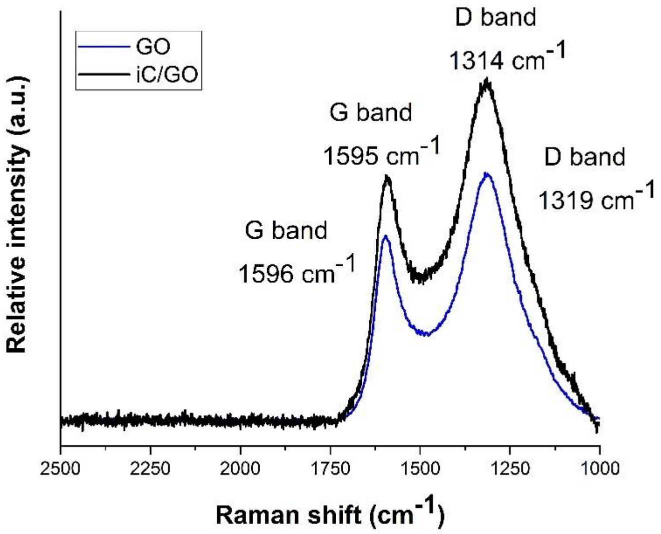

Figure S2. Raman spectra of GO and the GO (0.5% w/v) dispersed in ιC (2% w/v).

Figure S3: UV-Vis

GO dispersions in solutions of ιC (2% w/v), gelatin (1% w/v) and κC (1% w/v) were analyzed by UV-Vis. It is observed that no absorbance peaks appear in the investigated wavelength range, but only a shift of the baseline due to light scattering caused by the dispersed nanoparticles. This baseline displacement is associated to the concentration of dispersed nanoparticles. **Figure S3** compares the dispersion of GO in ιC (2%) at different times of ultrasonication, from which it is evident that the baseline shifts more the longer the ultrasound treatment time, indicating a higher concentration of dispersed GO. Consequently, 10 min of sonication was selected for the preparation of the hydrogels, as it produces nice dispersions without agglomerates even at high concentrations, allowing homogeneous distribution within the gel.

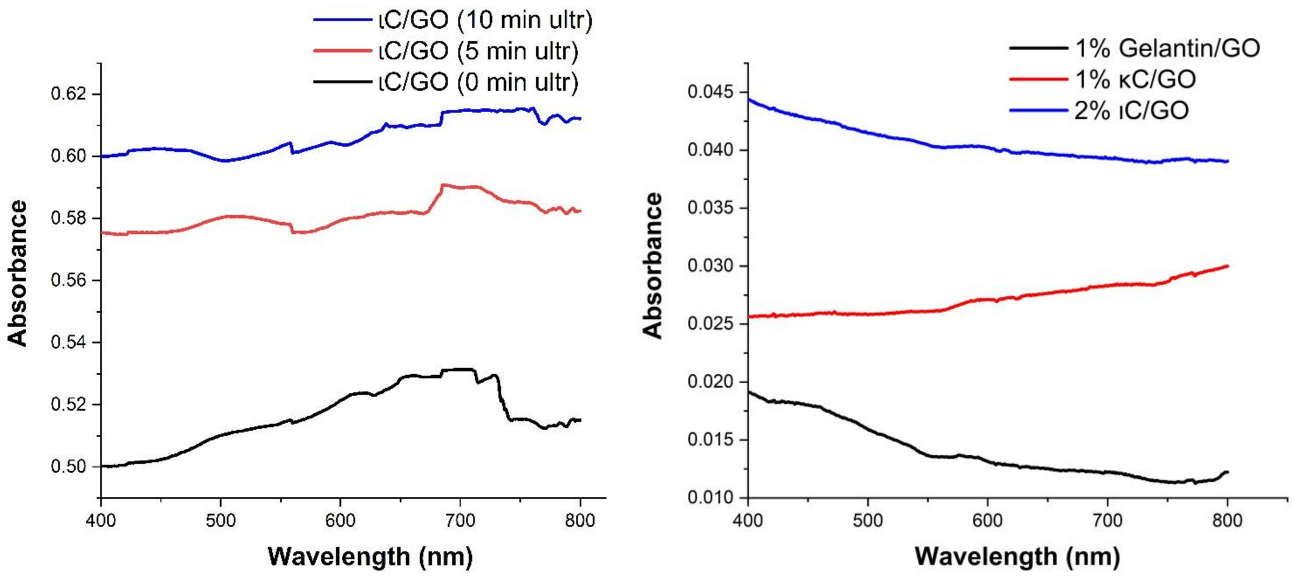

Figure S3. Spectra of the graphene (0.5%) suspension in ιC (2%) applying different ultrasonication times (5 min and 10 min).

Figure S4. X-Ray Diffraction

The X-ray patterns of the injectable hydrogels are shown in **Figure S4.**

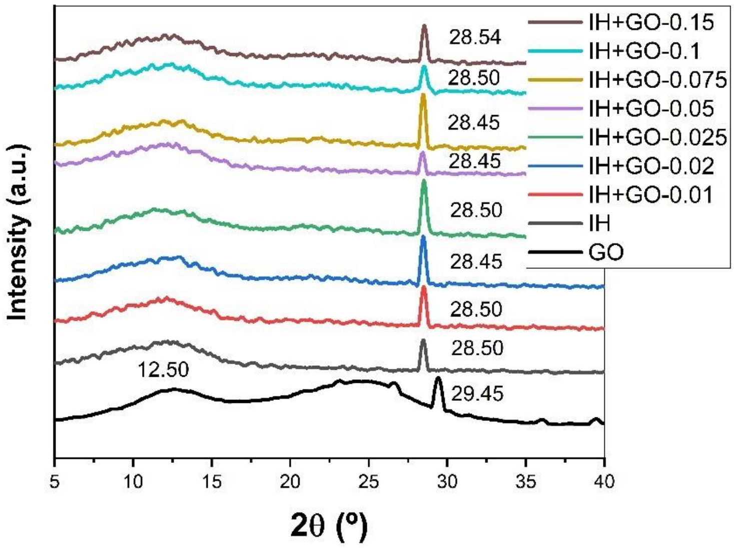

Figure S4. Analysis of XRD patterns of injectable hydrogels (IH/IH+GO).
